# Feasibility and dosimetric evaluation of single- and multi-isocentre stereotactic body radiation therapy for multiple liver metastases

**DOI:** 10.3389/fonc.2023.1144784

**Published:** 2023-04-28

**Authors:** Chunbo Tang, Changfei Gong, Biaoshui Liu, Hailiang Guo, Zhongyang Dai, Jun Yuan, Xiaoping Wang, Yun Zhang

**Affiliations:** ^1^ Department of Oncology, First Affiliated Hospital of Gannan Medical University, Ganzhou, China; ^2^ Department of Radiation Oncology, Jiangxi Cancer Hospital, Nanchang, China; ^3^ Department of Radiation Oncology, Sun Yat-sen University Cancer Center, Guangzhou, China

**Keywords:** RapidPlan, multiple liver metastases, stereotactic body radiotherapy, single-isocentre, normal tissue complications

## Abstract

**Objectives:**

Single-isocentre volumetric-modulated arc therapy (VMAT) stereotactic body radiation therapy (SBRT) improves treatment efficiency and patient compliance for patients with multiple liver metastases (MLM). However, the potential increase in dose spillage to normal liver tissue using a single-isocentre technique has not yet been studied. We comprehensively evaluated the quality of single- and multi-isocentre VMAT-SBRT for MLM and propose a RapidPlan-based automatic planning (AP) approach for MLM SBRT.

**Methods:**

A total of 30 patients with MLM (two or three lesions) were selected for this retrospective study. We manually replanned all patients treated with MLM SBRT by using the single-isocentre (MUS) and multi-isocentre (MUM) techniques. Then, we randomly selected 20 MUS and MUM plans for training to generate the single-isocentre RapidPlan model (RPS) and the multi-isocentre RapidPlan model (RPM). Finally, we used data from the remaining 10 patients to validate RPS and RPM.

**Results:**

Compared with MUS, MUM reduced the mean dose delivered to the right kidney by 0.3 Gy. The mean liver dose (MLD) was 2.3 Gy higher for MUS compared with MUM. However, the monitor units, delivery time, and V20Gy of normal liver (liver-gross tumour volume) for MUM were significantly higher than for MUS. Based on validation, RPS and RPM slightly improved the MLD, V20Gy, normal tissue complications, and dose sparing to the right and left kidneys and spinal cord compared with manual plans (MUS vs RPS and MUM vs RPM), but RPS and RPM significantly increased monitor units and delivery time.

**Conclusions:**

The single-isocentre VMAT-SBRT approach could be used for MLM to reduce treatment time and patient comfort at the cost of a small increase in the MLD. Compared with the manual plans, RapidPlan-based plans, especially RPS, have slightly improved quality.

## Introduction

Malignant tumors of the liver include primary hepatobiliary cancers and metastatic tumors. Liver metastases usually originate from colorectal cancer, lung cancer, pancreatic cancer, adenocarcinoma, breast cancer, and other malignant tumors (1). Surgical resection of liver metastases remains the primary treatment option. Unfortunately, only a small number of patients are suitable for surgery because of insufficient functional liver reserve and medical complications (2, 3). Alternative options include stereotactic body radiotherapy (SBRT), radiofrequency ablation, chemoembolization, and radioembolization. These techniques are promising in a considerable number of patients with liver metastases (4). SBRT is defined as an effective, non-invasive, and highly accurate hypo-fractionated radiotherapy technique that has been prospectively shown to provide a good local control rate (5). Several studies have studied the feasibility, safety, and clinical outcomes of SBRT for primary liver cancer and metastases (6–12).

Over the past few years, researchers have increasingly focused on the effectiveness of SBRT in treating systemic metastatic tumors and have analyzed the effect of dose distribution on tumor targets and organs at risk (OARs). Owing to the technical challenges inherent in treating multiple lesions as their spatial separation decreases, Hallaq et al. (13) investigated the rationale of technical requirements of SBRT for multiple metastases. Wang et al. (14) and Ruggieri et al. (15) conducted studies on brain metastasis with SBRT. They showed that extending the treatment time would increase the position error, resulting in a decrease in the accuracy of dose delivery. Based on a multi-institution study, Rusthoven et al. (16) demonstrated that high-dose liver SBRT is safe and effective for treating patients within three hepatic metastases. Clark et al. (17) evaluated the plan quality of single-isocentre versus multi-isocentre volumetric modulated arc therapy (VMAT) for multiple central nervous system metastases. The preliminary results indicated the delivery time of a single-isocentre was less than half of the multi-isocentre while maintaining comparable planning quality. Recently, with advances in optimization modalities and delivery techniques, dosimetric performance and therapeutic efficiency have been greatly improved for liver SBRT. By comparing the treatment plan quality, robustness, and plan complexity of robust optimization and the planning target volume (PTV)-based optimized plans, Miura et al. (18) found that robust optimization provides stable target coverage with shifted locations and helps to slightly reduce plan complexity for liver SBRT. Thaper et al. (19) studied whether the dynamic conformal arc integrated with the segment shape optimization and variable dose rate was superior to the classic VMAT. To harmonize liver SBRT practice and to fill the knowledge gap concerning the inter-system and inter-user differences for treatment techniques and treatment planning systems (TPS), Moustakis et al. (20) performed a meaningful study of planning benchmark for SBRT of liver metastases and provided the best practice guidelines for users. Although the aforementioned studies have studied the feasibility, safety and efficiency of liver SBRT, and have potential benefits in terms of solving some issues, to the best of our knowledge, none of the studies that investigated the dosimetric impact of single-isocentre SBRT for multiple liver metastases (MLM), and the potential increase in dose spillage to the normal liver tissue using a single-isocentre technique has not yet been studied. In this work, we aimed to investigate whether a single-isocentre technique leads to increased normal liver dose compared to a conventional multi-isocentre technique for MLM SBRT.

Technological advances in radiotherapy over the past decade have enabled the creation and delivery of smarter plans (21). Deliberately mimicking the behavior of experienced planners, automatic planning (AP) algorithms, including Auto-Planning (Philips Medical Systems, Best, The Netherlands) (22), RapidPlan (Varian Medical Systems, Palo Alto, CA) (23), and Multi-Criteria Optimization (RaySearch Laboratories, Stockholm, Sweden) (24), have been developed to accelerate the treatment planning process and drastically improve the planning efficiency. In particular, the knowledge‐based (KB) RapidPlan adopts machine learning or statistical methods to extract historical planning information and establishes a prediction model to estimate the expected dose-volume histograms (DVHs) for new patients (25–27). Many studies have emerged on the application of RapidPlan to various anatomical locations (28–30). Only two previous studies have evaluated the benefit of RapidPlan technology in the treatment of liver cancer. Gang et al. (31) constructed a special RapidPlan based on the distance between the right kidney and the PTV, as well as a general KB model and compared the prediction ability of the two models. Antonella Fogliata et al. (32) built another general KB model; the results showed the optimization engine can produce a clinically acceptable plan for liver cancer. However, there has yet to be a RapidPlan model to automate treatment planning for multi-lesion liver SBRT including deploying beam geometry and optimization. Based on these considerations, we also evaluated RapidPlan for MLM SBRT.

More specifically, our major contributions include the following. First, we verified the technical feasibility of single- and multi-isocentre for MLM SBRT and performed the relevant dosimetric evaluation. Second, we extended the RapidPlan model for MLM by incorporating SBRT and compared it with manual planning. Finally, we comprehensively evaluated these four approaches – that is, the manual single-isocentre technique (MUS), the manual multi-isocentre technique (MUM), the RapidPlan single-isocentre technique (RPS), and the RapidPlan multi-isocentre technique (RPM).

## Materials and methods

### Patients and target delineation

We selected 30 patients with MLM (two or three lesions) treated in our hospital between August 2020 and February 2022 for this retrospective study. All patients were immobilized using a stereotactic body frame with a vacuum fixation cushion to create a reproducible position. Four-dimensional computed tomography (4DCT) (CT0, CT1…CT9) was acquired with 2 mm slice thickness using Siemens Medical Systems and transferred to the Eclipse TPS (Version 15.5). The gross tumor volumes (GTVs) were contoured on 4DCT by one senior radiation oncologist according international guidelines (20, 33). The internal target volumes (ITVs) encompassing the whole respiratory tumor motion areas were generated after extension of GTVs and the final PTV was generated by uniformly expanding ITVs with a 5 mm margin. Critical structures including the normal liver, heart, spinal cord, and kidneys were also contoured. **Table 1** lists the detailed clinical characteristics of these patients.

**Table 1:** Characteristics of the 30 patients with MLM enrolled in this study.

Number of patients	30
Gender	
Male	22(73.3%)
Female	8(26.7%)
Age (years)	
Median [range]	59[38-84]
Mean ± SD	58.4±11.8
Location	
Left lobe	8(26.7%)
Right lobe	17(56.7%)
Both sides	5(16.6%)
Number of lesions	
Two	25(83.3%)
Three	5(16.7%)
PTV volume [cm^3^]	
Median [range]	56.9[9.9-153.9]
Mean ± SD	68.1±48.7
Primary tumor	
Breast cancer	3(10%)
Lung cancer	6(20%)
Rectum cancer	12(40%)
Cervix cancer	5(16.7%)
Colon cancer	4(13.3%)
Normal liver volume [cm^3^]	
Median [range]	1090[691-1477]
Mean ± SD	1091.3±187.0
**Prescription dose (Gy)**	56

### Treatment planning

The prescribed dose was 56 Gy in 7 fractions for all patients. The machine optimization settings were as follows: 6 MV FFF beam with dose rate 1400 MU/min, AcurosXB with dose-to-water reporting mode and PO dose calculation algorithms with heterogeneity corrections with 2.0 × 2.0 × 2.0 mm calculation grid size, and the jaw tracking was activated during optimization to reduce MLC transmission or leakage (34). For MUM and RPM, multiple isocentres were placed at the center of each lesion, and 2-4 partial coplanar arcs with 30° collimator angles were utilized to reduce the MLC leakage dose. For MUS and RPS, all lesions were integrated into a single target area with a maximum inter-target distance of less than 15 cm, and then the single isocentre was placed in the geometric center of this target. The optimal setting of two partial coplanar arcs was adopted with a 30° collimator angle. All treatment plans were made by one senior medical physicist to avoid inter-planner variability.

For manual planning, all 30 patients treated with SBRT were retrospectively replanned using MUS and MUM. Based on clinical requirements, experience, and customization, we pre-defined the initial optimization objectives. To ensure appropriate dose coverage and homogeneity, we optimized all plans, giving great weight for the PTV and less weight for OARs based on the as low as reasonably achievable (ALARA) concept (20). Typically, to achieve acceptable SBRT plans, the planner needs to introduce auxiliary structures frequently and adjust the constraints repeatedly. We utilized dose-limiting shells and normal tissue objectives (NTOs) to limit hot spots or to control dose fall-off outside the PTV. Based on previous studies and SBRT/IGRT protocols (20, 33), at least 98% of the PTV received 100% of the prescribed dose, conformity index (CI) less than 1.2 is desirable, D2cm (maximum dose at any point 2 cm away from the PTV margin in any direction) has to be smaller than 50–70%, depending on the PTV size. The maximum dose to the PTV was planned fell inside the GTVs. We also considered vulnerable OARs constraints for evaluation of the mean liver dose (MLD); V20Gy of normal liver (liver-GTVs); dose to 0.5 cc (D0.5cc) of the stomach, esophagus, and small bowel; maximum dose (Dmax) to the planning risk volume (PRV) to the spinal cord (plus a 0.5 cm margin); and mean dose (Dmean) to the left and right kidneys. We performed multiple re-optimizations to meet the final clinical requirements. The settings of optimization goals and priority are in **Table S1** (**supplementary material**).

**Figure 1:**
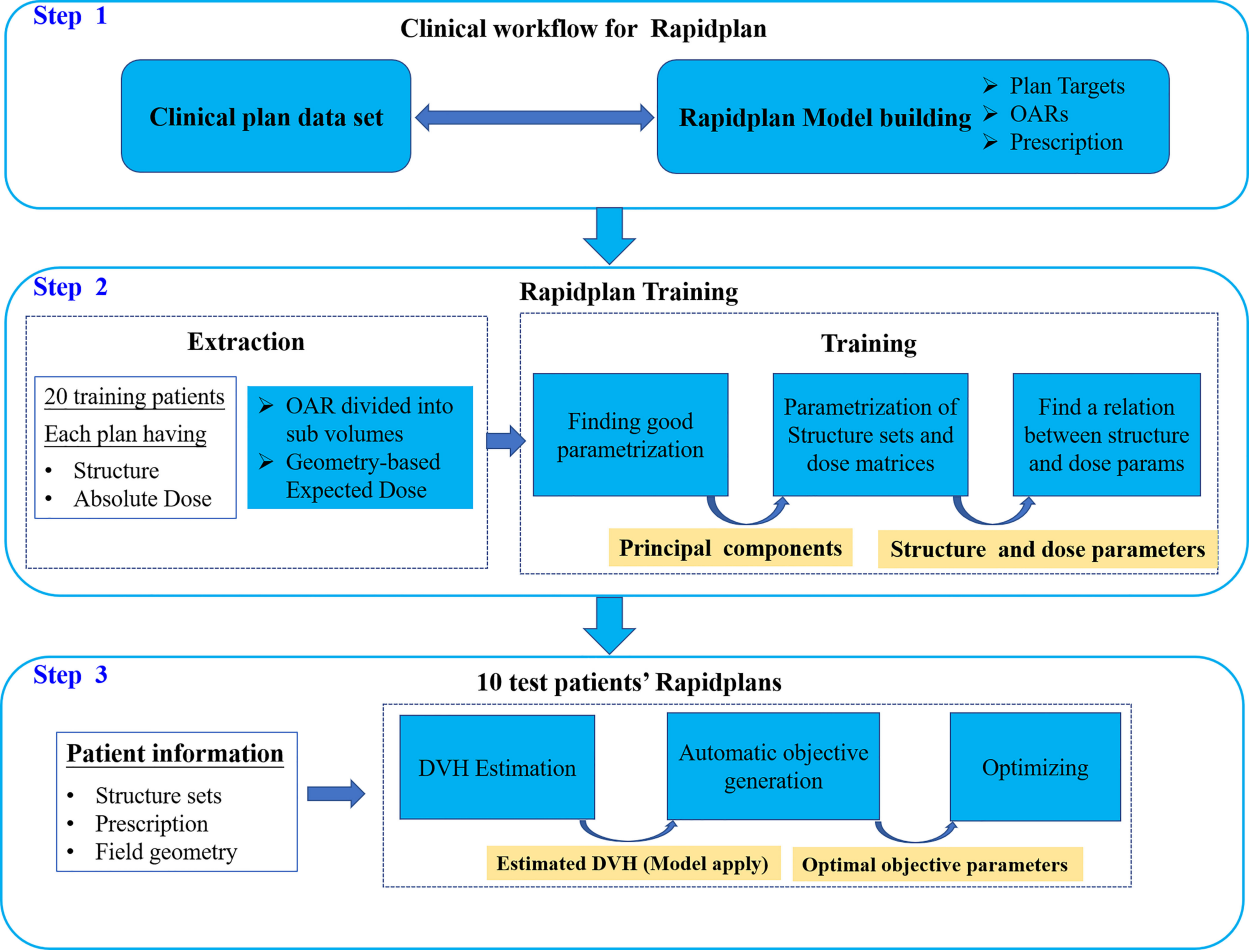
Schematic of the workflow for RapidPlan modelling.

For RapidPlan, we randomly selected 20 MUS and 20 MUM for training to generate RPS and RPM models, respectively. **Figure 1** shows the clinical workflow of RapidPlan modelling and training steps. The RapidPlan optimization component consists of three main parts: the modelling and training engine, the automatic constraint prediction module, and new VMAT/IMRT optimization functions. The main steps for modelling and training were: (1) we selected the clinically accepted cases and created the unified RapidPlan model. (2) We defined the structures, optimization objectives, initial constraints, and priorities of the model. (3) We added each training case to the model and associated the model structure with the prescription and optimization goal of the plan. (4) We extracted the dose and geometric feature information by using the principal component analysis method (35, 36). (5) We trained and finalized the model. For testing, based on the published RPS and RPM models, we optimized 10 RPS and 10 RPM plans that were not included in the training dataset. The main steps of validation were: (1) we selected the corresponding RPS or RPM model. (2) We manually matched the validation plan structure to the model structure. (3) We generated the DVH estimation range of the relevant constraint structures by the sophisticated regression model and automatically generating the dose-volume constraints. (4) We optimized the verification plan.

### Plan evaluation and analysis

We calculated and compared the following dosimetric evaluation metrics: Dmean and Dmax (the mean dose and maximum dose to target volume), D2cm, D2, D95, D98, and V100% (Vx represents the volume receiving at least x% of the prescription dose) of the PTV. We evaluated the gradient index (GI) (V50%/V100%) and the CI, and performed DVH analysis for each plan (37–40). The lower the GI value, the steeper the dose gradient. We defined the CI as the ratio between the PTV covered by the prescribed dose and the PTV. A CI value closer to 1 indicates better conformity.

We evaluated the MLD and V20Gy for normal liver (liver-GTVs) and analyzed the potential relationships between the MLD, the ITV, and the inter-target distance by linear regression analysis (34, 41–44). We assessed the probability of normal tissue complications (NTCPs) defined as the occurrence of radiation-induced hepatitis with liver enzyme changes grade ≥ 2 based on the MLD (EQD2) (42, 45, 46) for all treatment plans. Dmax to the spinal cord and the Dmean to kidneys were also evaluated. Additionally, we calculated the planned monitor units (MUs) and delivery time (beam-on time) to assess efficiency. Wilcoxon’s signed-rank test was carried out to calculate the statistical significance at a 95% confidence interval to compare different techniques and Bonferroni correction was used in order to account for multiple hypothesis testing. A p < 0.05 was considered statistically significant.

## Results

For the 30 cases, we generated 80 treatment plans (30 MUS, 30 MUM, 10 RPS and 10 RPM plans) for MLM SBRT. **Table 2** shows the results of MUS and MUM for the 30 patients: the two approaches achieved good dosimetry goals and similar target performance. Compared with MUS, MUM had a lower D2cm (28.58 ± 3.85) and reduced the mean dose delivered to the right kidney by 0.3 Gy. The mean dose to the left kidney (1.2 Gy) and the maximum dose to the spinal cord (14.2 Gy) of MUM were similar to MUS. MUS increased the mean dose of MLD by 2.3 Gy compared with MUM. However, the MUs, delivery time, and V20Gy of MUM were significantly higher than those of MUS.

**Table 2:** Quantitative evaluation of MUS and MUM for the 30 patients.

DVH metrics		MUS	MUM	p
PTV Dmean (Gy)	Median [range]	62.5[61.4-64.2]	62.9[61.9-65.5]	
	Mean ± SD	62.6 ± 0.7	63 ± 0.8	0.002
PTV D2% (Gy)	Median [range]	67.4[66.7-71.6]	67.7[37.3-72.6]	
	Mean ± SD	67.7 ± 1.0	66.8 ± 6.4	0.019
PTV D95% (Gy)	Median [range]	56.9[56.1-58.7]	57.1[55.8-58.9]	
	Mean ± SD	57.1 ± 0.7	57.1 ± 0.7	0.540
PTV D98% (Gy)	Median [range]	55.8[54.8-57.7]	55.9[55.0-58.0]	
	Mean ± SD	55.9 ± 0.8	56 ± 0.8	0.410
PTV Dmax (Gy)	Median [range]	69.1[67.8-70.9]	69.5[67.8-73.9]	
	Mean ± SD	69.3 ± 1.1	69.8 ±1.5	0.030
PTV D100% (%)	Median [range]	98.0[95.4-99.8]	97.9[89.1-99.9]	
	Mean ± SD	97.9 ±1.45	97.7 ± 2.2	0.440
CI	Median [range]	1.01[0.97-1.12]	1.03[0.93-1.19]	
	Mean ± SD	1.03 ± 0.04	1.04 ± 0.06	0.002
GI	Median [range]	3.9[3.2-5.3]	3.9[3.1-4.4]	
	Mean ± SD	4.0 ± 0.6	3.8 ± 0.4	0.001
D2cm (Gy)	Median [range]	30.0[23.63-43.04]	29.58[20.0-34.72]	
	Mean ± SD	29.67±4.18	28.58±3.85	0.304
MUs	Median [range]	3200[1993-4236]	4368[3608-6953]	
	Mean ± SD	3243 ± 572	4377 ± 728	0.001
Delivery time (minutes)	Median [range]	2.8[1.9-4.1]	3.8[3.1-6.9]	
	Mean ± SD	3.0 ± 0.6	3.9 ± 0.8	0.001
MLD (Gy)	Median [range]	13.7[8.2-23.1]	13.7[6.6-21.6]	
	Mean ± SD	15.3 ± 3.9	13.0 ± 4.1	0.001
V20Gy (cc)	Median [range]	679[196.5-977]	722.5[306.5-1009]	
	Mean ± SD	675 ± 188.9	713.5 ± 185.4	0.001
Kidney-R Dmean (Gy)	Median [range]	1.7[0.2-8.9]	1.6[0.2-7.3]	
	Mean ± SD	2.1 ± 2.1	1.8 ± 1.7	0.08
Kidney-L Dmean (Gy)	Median [range]	0.6[0.08]	0.5[0.07-4.4]	
	Mean ± SD	1.2 ± 1.1	1.2 ± 1.1	0.930
Spinal cord Dmax (Gy)	Median [range]	15.2[1.0-25.6]	13.0[1.0-26.1]	
	Mean ± SD	14.2 ± 6.3	14.2 ± 5.7	0.051

PTV, planning target volume; MLD, mean liver dose; CI, manually plans; GI, gradient index; MUs, monitor units; MUS, manual-based single-isocentre technique; MUM, manual-based multi-isocentre technique.

**Figure 2:**
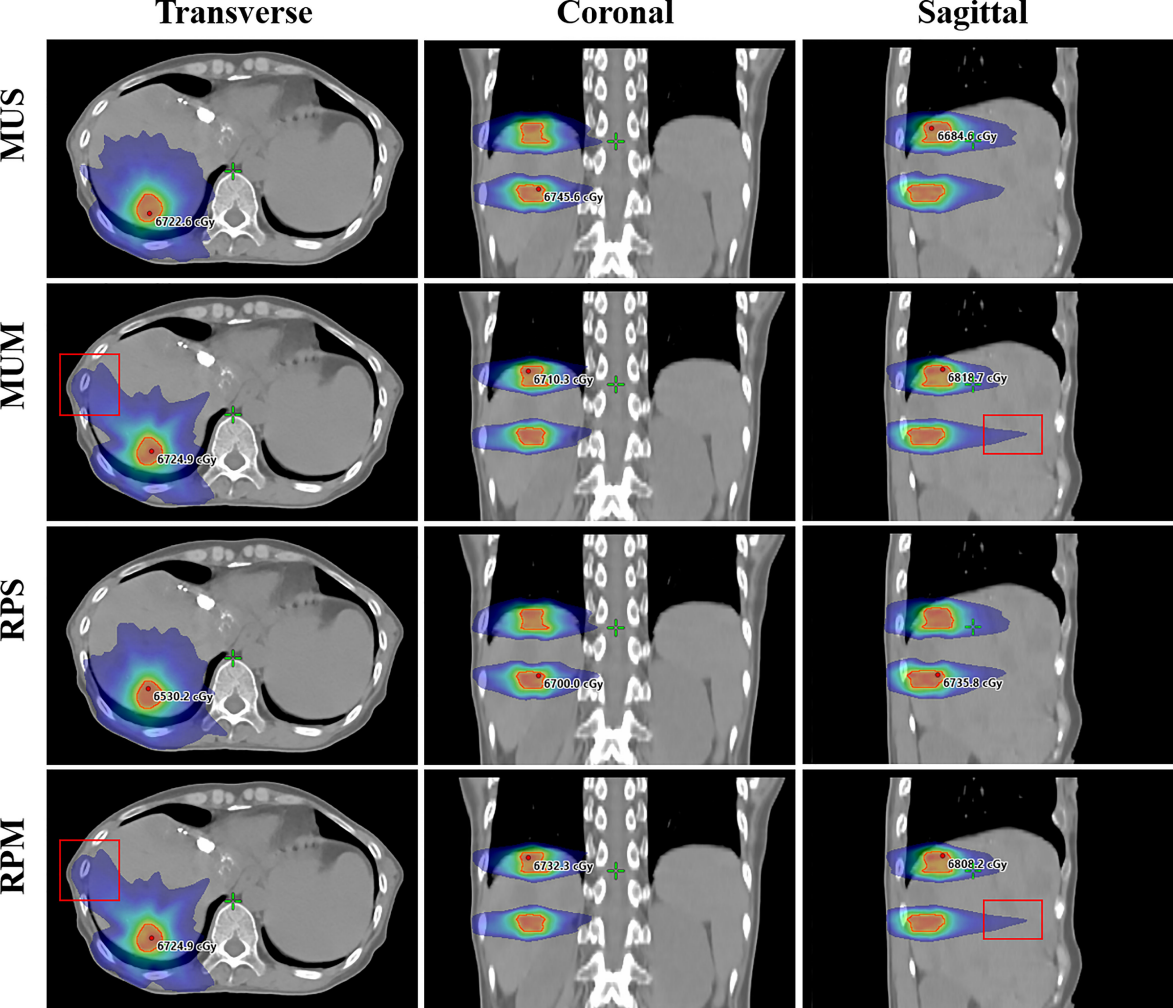
Comparison of transverse, coronal, and sagittal dose distributions for one example case.

**Figure 3:**
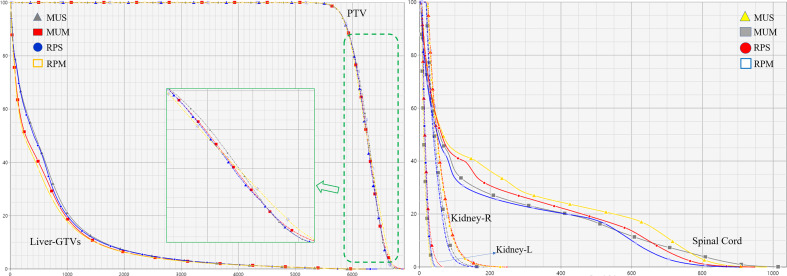
Comparison of DVHs for the PTV and OARs.

**Figure 2** illustrates the transverse, coronal, and sagittal dose distributions for different regions of a representative patient and the corresponding cumulative DVHs for the PTV and OARs is shown in **Figure 3**. In the visual inspection, the isodose lines are more conformal and constricted to the PTV for RPS and MUS compared with RPM and MUM. Especially for the transverse and sagittal views, there is greater dose spill over as indicated by the red square in **Figure 2**, which shows a steeper dose gradient and improved conformity for single-isocentre plans (RPS and MUS). The detailed dosimetric differences of the target volume and individual organs are presented as DVHs for this patient in **Figure 3**.

**Figure 4:**
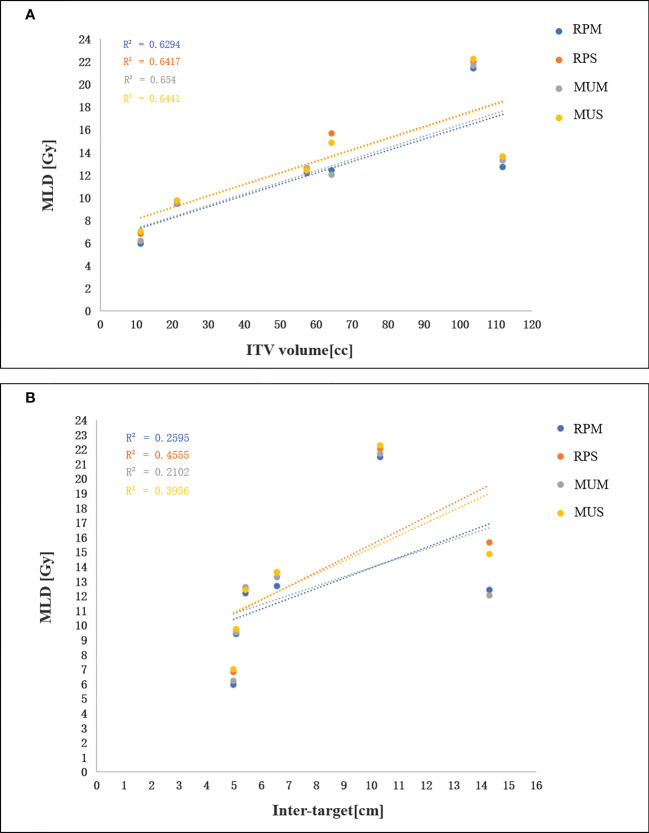
**(A)** Linear regression analysis between the MLD and the ITV. **(B)** linear regression analysis between the MLD and inter-target distance.

**Table 3** shows an overview of the numerical findings of MUS, MUM, RPS, and RPM based on average DVH analysis of the PTV and OARs for the 10 patients. There were no significant differences for the PTV metrics except for D2 and Dmax, while the quality of D2cm and the GI for MUS (28.5 ± 2.1) and RPS (28.4± 3.2) was lower than that of MUM and RPM, and RPS (69.0 ± 1.1) had the best hotspot control. RPM had the lowest MLD and it was significantly better than those of RPS and MUS (p < 0.05). On the contrary, V20Gy of liver GTVs for RPM and MUM was larger than that of MUS and RPS. Overall, for the MLD, V20Gy, NTCPs, and dose sparing to the left and right kidneys and the spinal cord, AP plans showed slight improvements compared with manual plans (MUS vs RPS, MUM vs RPM), but the AP plans significantly increased MUs and delivery time. Linear regression showed no significant correlation between the MLD and the inter-target distance (p > 0.05) (**Figure 4A**). There was a significant correlation between the MLD and the ITV for all approaches (p < 0.05) (**Figure 4B**).

**Table 3:** Quantitative evaluation on the average, standard deviation, and range of DVH metrics for the PTV and OARs among MUS, MUM, RPS, and RPM.

Structures	DVH Metrics	MUS	MUM	RPS	RPM	Pairwise comparison
PTV	Dmean (Gy)	62.6±0.6	62.6±0.5	62.4±0.5	62.7±0.3	i=0.046,ii=0.6,iii=0.612,
						iv=0.047,v=0.249,vi=0.5
	D_2_ (Gy)	67.4±0.6	67.8±0.7	67.1±0.5	67.9±0.6	i=0.028,ii=0.502,iii=0.173
						iv=0.042,v=0.028,vi=0.075
	D_95_ (Gy)	57.4±0.8	57.4±0.8	57.1±1.1	57.2±0.9	i=0.249,ii=0.116,iii=0.075,
						iv=0.246,v=0.116,vi=0.601
	D_98_ (Gy)	56.2±1.0	56.2±1.0	55.8±1.3	55.9±1.1	i=0.345,ii=0.249,iii=0.116,
						iv=0.249,v=0.116,vi=0.463
	Dmax(Gy)	69.2±0.6	70.7±1.8	69.0±1.1	70.9±2.2	i=0.026,ii=0.753,iii=0.116,
						iv=0.029,v=0.344,vi=0.028
	V100(%)	98.3±1.1	98.7±0.7	97.2±1.9	97.58±0.7	i=0.042,ii=0.248,iii=0.223,
						iv=0.093,v=0.173,vi=0.395
	CI	1.04±0.06	1.05±0.06	1.03±0.07	1.03±0.06	i=0.225,ii=0.116,iii=0.172,
						iv=0.08,v=0.080,vi=0.248
	GI	3.7±0.63	3.8±0.53	3.6± 0.58	3.7± 0.47	i=0.172,ii=0.753,iii=0.463,
						iv=0.461,v=0.917,vi=0.917
	D2cm (Gy)	28.5±2.1	28.7±2.6	28.4± 3.2	29.3± 3.1	i=0.075,ii=345,iii=0.174,
						iv=0.611,v=0.917,vi=0.247
Liver-GTVs	MLD(Gy)	13.5± 5.2	12.6± 5.2	13.4±5.3	12.4±5.2	i=0.028,ii=0.172,iii=0.029,
						iv=0.044,v=0.916,vi=0.076
	V20Gy (cc)	715±250	749±248	708±226	748±228	i=0.027,ii=0.602,iii=0.027,
						iv=0.046,v=0.402,vi=0.172
Kidney_L	Dmean (Gy)	2.2±1.4	2.1±1.3	1.9±1.2	1.8±1.3	i=0.614,ii=0.345,iii=0.118,
						iv=0.600,v=0.173,vi=0.463
Kidney_R	Dmean (Gy)	5.9±5.2	5.9±4.8	4.3±3.7	4.3±3.9	i=0.334,ii=0.170,iii=0.115,
						iv=0.344,v=0.115,vi=0.025
SpinalCord	Dmax (Gy)	12.2±3.8	12.1±3.6	11.8±3.9	10.8±3.6	i=0.249,ii=0.119,iii=0.075,
						iv=0.753,v=0.463,vi=0.461
NTCPs	(%)	42.3±3.2	41.8±3.2	42.1±3.2	41.7±3.2	i=0.028,ii=0.173,iii=0.026,
						iv=0.116,v=0.462,vi=0.046
Monitor units	(MUs)	3366±581	5453±1489	3785± 1193	5597± 1658	i=0.028,ii=0.463,iii=0.281,
						iv=0.028,v=0.463,vi=0.028
Delivery time	(minutes)	3.0±0.4	4.8±1.2	3.1±0.6	4.9±1.3	i=0.029,ii=0.345,iii=0.028,
						iv=0.027,v=0.345,vi=0.027

PTV, planning target volume; MLD, mean liver dose; CI, manually plans; GI, gradient index; NTCPs, normal tissue complications; MUS, manual-based single-isocentre technique; MUM, manual-based multi-isocentre technique; RPS, RapidPlan-based single-isocentre technique; RPM, RapidPlan-based multi-isocentre technique; i, RPS vs RPM; ii, RPM vs. MUM; iii, RPM vs. MUS; iv, RPS vs. MUM; v, RPS vs. MUS; vi, MUM vs. MUS.

## Discussion

Single-isocentre SBRT for MLM is an emerging technique for medically inoperable patients (6–9). However, there are little data about the dosimetric impact of single-isocentre SBRT for MLM compared with a conventional multi-isocentre SBRT technique, and the potential increase in dose spillage to the normal liver tissue using a single-isocentre technique. So, we conducted a comprehensive comparison between single- and multi-isocentre techniques and investigated whether a single-isocentre technique leads to increased normal liver dose for MLM SBRT. First, we manually designed the single- and multi-isocentre plans for all 30 patients. The two approaches achieved good dosimetry goals, as shown in **Table 2**. Compared with the single-isocentre technology, the multi-isocentre technology plans slightly improved the PTV, OARs sparing, and the MLD. On the other hand, the V20Gy, MUs, and delivery time of multi-isocentre plans were significantly higher than those of single-isocentre plans, and we found that these indicators were larger in patients with three lesions (5 patients) compared with patients with two lesions (25 patients). These results are consistent with recent studies that compared the dosimetric quality of single-isocentre multi-lesion lung SBRT with the multi-isocentre technique (47). Single-isocentre SBRT for MLM could be safely used to reduce position error and delivery time and to improve cost-effectiveness, even though we observed a relevant increase in the MLD.

Despite growing interest in single-isocentre/multiple-lesion VMAT SBRT treatments, difficulties due to complex multi-lesion SBRT planning have been described (13, 20, 47–49). When treating multiple lesions with a single- or multi-isocentre VMAT plan, the beam fields in the target interval are likely to interact with each other, and it is difficult to control the dose spill over outside the target area. Currently, manual treatment planning is still the most utilized approach in clinical practice, whereas assigning parameters, tweaking constraints, and setting weights remains labor intensive. During the optimization process, dose-limiting shells, and regions of interest of hot/cold spots are frequently introduced to achieve a proper balance between target coverage and OARs sparing. Due to the complexity of MLM and variable skills among planners, one of the major challenges of VMAT-SBRT planning is the large variation in the quality of plans. With limited clinical resources and time, even senior physicists may not be able to get the expected MLM plan. Manual planning depends heavily on the experience of the planner. Moreover, it is impossible for physicians/dosimetrists to know whether optimization attempts have minimized the OARs doses to the lowest attainable level within an acceptable time frame. Trying to overcome the limitations of MLM SBRT planning, we have presented a novel implementation of the multi-isocentre AP approach for MLM SBRT alongside its operability and advantages with respect to previously manually designed plans in terms of planning quality and efficiency.

Based on the manually optimized single-/multi-isocentre plans and in line with the clinical requirements, we constructed the general single- and multi-isocentre AP models. For either single- or multi-isocentre plans, the AP plans are higher quality than the manual plans, although there were no significant differences in some PTV metrics. Based on OARs sparing and the probability of liver NTCPs, the AP plans, especially RPS, are also better than the manual plans. We showed that RPS and RPM improve sparing of the spinal cord and kidneys in the max and medium dose range compared with MUS and MUM (see **Table 3**). In our study, the MUs of single-isocentre plans decreased by 62% (MUS vs MUM) and 40% (RPS vs RPM) compared with multi-isocentre plans. Furthermore, the results of 30 cases of manual planning and 10 cases of AP showed a significant correlation between the MLD and the ITV for all techniques, but the MLD was not correlated with the distance between lesions. Many factors affect the quality of AP, but the main factors are the quality of the plan in the model library and the geometry of the target area. To ensure the consistency of the target structure and the efficiency of the model, we established two general training models. Our study can provide some guidance for the clinical application of multiple metastases of hepatocellular carcinoma.

There are some potential limitations to this work. First, although we enrolled 30 patients with MLM, due to the inherent requirements of the model training set, the number of test patients is relatively small. Since we only compared treatment plans and the single-isocentre technique has not yet been widely clinically applied for MLM SBRT, and our conclusions could be further supported by increasing the sample size in future studies. Second, dose/plan verification is not investigated in the current retrospective study, and this should be done before clinical radiotherapy. However, we consider these limitations to be of minimal importance and they have not significantly influenced the overall findings of the study.

## Conclusion

We have shown the feasibility of single-/multi-isocentre SBRT using RapidPlan technique for MLM and have presented the resulting dosimetric impact for single- versus multi-isocentre MLM SBRT plans. One could use a single-isocentre approach for MLM to reduce treatment time and patient comfort at the cost of a small increase in the MLD. Compared with the manual plans, the RapidPlan-based plans slightly improve the PTV, OARs, and the MLD at the cost of increased MUs and delivery time.

## Data availability statement

The original contributions presented in the study are included in the article/**Supplementary Material**. Further inquiries can be directed to the corresponding authors.

## Ethics statement

The studies involving human participants were reviewed and approved by the Institution Review Board and Ethics Committee of Jiangxi Cancer Hospital. Written informed consent to participate in this study was provided by the participants’ legal guardian/next of kin.

## Author contributions

CT: data curation, methodology, project administration, writing – original draft. CG: conceptualization, formal analysis, writing – review, and editing. BL: data curation, formal analysis. HG: formal analysis, and investigation. ZD: investigation, project administration. JY: data curation, formal analysis. XW: data curation, and investigation. YZ: conceptualization, formal analysis, writing – review, and editing. All authors contributed to the article and approved the submitted version.
