# Supplementary material for: Feasibility and dosimetric evaluation of single- and multi-isocentre stereotactic body radiation therapy for multiple liver metastases
Source: Front Oncol. 2023 Apr 28;13:1144784. doi: 10.3389/fonc.2023.1144784 (PMC10175834; doi:10.3389/fonc.2023.1144784)
Supplement: Supplementary file 1 [file Table_1.docx]

| **ROIs** | **Type** | **Dose (cGy)** | **Volume max** | **Priority** |
| --- | --- | --- | --- | --- |
| GTV | Max Dose | 6700 | 0% | 200 |
| GTV | Lower DVH | 5600 | 99% | 210 |
| PTV | Max Dose | 6600 | 0% | 200 |
| PTV | Lower DVH | 5700 | 98% | 210 |
| PTV | Lower DVH | 5600 | 99% | 230 |
| Esophagus | Max Dose | 2000-3000 |  | 180 |
| Esophagus | Max DVH | 2200-3200 | 5% | 180 |
| Ring1 | Max Dose | 5600 |  | 160 |
| Ring2 | Max Dose | 3200 |  | 180 |
| Ring3 | Max Dose | 2400 |  | 190 |
| Heart | Max Dose | 600-1200 |  | 160 |
| Heart | Max DVH | 800-1400 | 2% | 160 |
| Kidney-L | Mean Dose | 500-700 |  | 100 |
| Kidney-R | Mean Dose | 500-800 |  | 100 |
| Liver-GTVs | Mean Dose | 1200-1600 |  | 200 |
| Ribs | Max Dose | 3000-4000 |  | 180 |
| Spinal Cord | Max Dose | 1000-2300 |  | 180 |
| Stomach | Max Dose | 1600-2500 |  | 180 |
| Stomach | Max DVH | 1800-2500 | 10% | 180 |
| Small bowels | Max Dose | 1300-2400 |  | 160 |
| Small bowels | Max DVH | 1600-2240 | 0.5% | 160 |

Table S1 Optimization parameters in MUS and MUM
